# A New Anthropomorphic Mannequin for Efficacy Evaluation of Thoracic Protective Equipment Against Blast Threats

**DOI:** 10.3389/fbioe.2021.786881

**Published:** 2022-01-27

**Authors:** Johanna Boutillier, Venetia Cardona, Pascal Magnan, Michael Ogier, Sébastien De Mezzo, Florent Montespan, William Menini, Joël Mosnier, Pierre Naz, Nicolas J. Prat

**Affiliations:** ^1^ French-German Research Institute of Saint-Louis (ISL), Saint-Louis, France; ^2^ French Armed Forces Biomedical Research Institute (IRBA), Brétigny sur Orge, France; ^3^ French Military Training Hospital Saint-Anne, Toulon, France

**Keywords:** mannequin, swine, thoracic protective equipment, blast, lung injury

## Abstract

Exposure to blast is one of the major causes of death and disability in recent military conflicts. Therefore, it is crucial to evaluate the protective capability of the ballistic-proof equipment worn by soldiers against the effects of blast overpressure (i.e., primary blast injuries). A focus will be made on thoracic protective equipment (TPE). An anthropomorphic mannequin, called BOPMAN, and anesthetized swine both wearing soft, hard or no ballistic protection, were subjected to an open-field high-intensity blast. For swine, thoracic wall motion (acceleration and velocity) was recorded during blast exposure and severity of lung injury was evaluated postmortem. Different data were collected from BOPMAN thoracic responses, including reflected and internal pressure, as well as the force at the rear face of the instrumented part. The severity of blast-induced lung injuries (contusion extent, Axelsson Severity Scale) and the thoracic wall motion were decreased in animals protected with thoracic ceramic hard plates as compared to those wearing soft or no protection. There was a clear trend towards greater lung injury in animals protected with the soft body armor used, even when compared to unprotected animals. In line with these experimental data, the measured force as well as the force impulse measured using BOPMAN were also decreased with a ceramic hard plate protection and increased when a soft ballistic pack was used compared to no protection. Comparison of data collected on BOPMAN and swine equipped with the same protection level revealed that those two force parameters were well correlated with the level of blast-induced lung injury (force, R^2^ = 0.74 and force impulse, R^2^ = 0.77, *p* < 0.05). Taken together, our results suggest that the force and the force impulse data from BOPMAN may help estimate the efficiency of existing TPE regarding lung protection under blast exposure and may represent an important tool for development of future TPE.

## 1 Introduction

Performance level of protective equipment for soldiers and law enforcement officers is usually evaluated and set against ballistic, stab and shrapnel threats, but not against blast overpressure, which represents a real threat in modern armed conflicts. Air-filled organs such as the lung, ears and gastrointestinal tract are particularly susceptible to primary blast. So far, little is known about the efficiency of protective equipment against blast-induced thoracic damage. Nevertheless, few studies have demonstrated that wearing thoracic protective equipment (TPE) worsens the level of blast-induced body injury, depending on the equipment used (Phillips et al., 1988; Cooper et al., 1991; Gibson, 1994; Cooper, 1996; Prat et al., 2019), although this finding seems to be inconsistent across studies (Long et al., 2009; Wood et al., 2013). Phillips et al. (1988) and Gibson (1994) have shown that multiple plies of ballistic fabric can amplify the peak pressure of the transmitted blast wave to the body. Higher extent of lung injuries was also observed in animals wearing low impedance materials such as ballistic fabric compared with unprotected animals (Phillips et al., 1988; Cooper et al., 1991). However, placing a high density material (such as a ceramic plate) between the low impedance material and the incoming blast wave may help reducing blast-induced lung injury or mortality rate (Cooper et al., 1991; Cooper, 1996; Sekine et al., 2021).

The primary blast threat has not been considered in the development of protection systems to be used by soldiers and law enforcement personnel so far, mainly because no specification exists. In order to correctly evaluate the performance of existing and future TPE against shock-waves produced by detonations of improvised explosive devices (IED), studies on thoracic models, especially mannequins, have recently emerged (Jetté et al., 2004; Bass et al., 2005; Ouellet and Williams, 2008; Carboni et al., 2010; Awoukeng Goumtcha et al., 2014). So far, the aim of these studies has been to demonstrate that the response of thoracic models is influenced by the TPE, although with this approach, one can only test if a protection system is better or worse than a reference system, without getting any information on the severity level of lung injury. The thoracic wall peak acceleration from a specific torso rig or the MABIL (“Mannequin for the Assessment of Blast Incapacitation and Lethality”) (Cooper et al., 1996b; Jetté et al., 2004; Ouellet and Williams, 2008) were found to be suitable for such an evaluation. For the Hybrid III mannequin, it was the thoracic reflected overpressure and the chest acceleration (Jetté et al., 2004; Bass et al., 2005; Carboni et al., 2010).

Comparing the efficacy of different TPE using thoracic surrogates is a real progress in the process of designing optimal protections, but evaluating the level of protection they offer regarding the severity of lung injury would be more appropriate and informative. Indeed, such an evaluation could help find a good compromise between the weight of the systems and their ability to protect. Unfortunately, to evaluate the level of protection of TPE regarding the injury risk, an adapted injury criterion is needed. Different injury criteria have been defined based on blast wave characteristics (Bowen et al., 1968; Bass et al., 2008), maximum thoracic wall velocity (Axelsson and Yelverton, 1996), or the normalized work (Stuhmiller et al., 1996). Incident pressure or impulse-based criteria are limited to unprotected scenarios. Indeed, whatever the equipment worn, the incident blast wave will be the same, and, therefore, criteria based on blast wave characteristics cannot be used to evaluate the impact of wearing TPE on the risk of injury (Boutillier et al., 2016). Regarding the maximum chest wall velocity or the normalized work, pressure measurements from each facet of the Blast Test Devices behind the TPE are needed. Those pressure profiles would then be applied on their respective mathematical thorax model to extract the estimated lung injury severity. In practice, there is no limitation to use those criteria when evaluating the efficacy of a TPE. Nevertheless, some issues with the validity of the criteria, regardless of the use of protection system, have been described (Boutillier et al., 2016). Moreover, no validation of the determined injury risk was performed with experimental data when using a TPE, which would limits the use of those models to unprotected scenarios.

The objective of this study was to demonstrate the ability of the new mannequin called BOPMAN (for “Blast OverPressure MANnequin”), specifically designed to model primary blast exposure, to estimate the risk for lung injury in protected, and unprotected soldiers. In this study, we correlated data from BOPMAN to the severity of lung injury measured in blast-exposed live animals. Anesthetized swine wearing soft, hard or no ballistic protection were subjected to an open-field, right sided, high-intensity blast, while the homemade anthropomorphic mannequin BOPMAN was exposed in a front standing position to the same threat. Effect of TPE on animal’s injury severity, thoracic wall motion and on BOPMAN thoracic response was investigated.

## 2 Materials and Methods

### 2.1 Animals

#### 2.1.1 Animals Preparation

Forty-one anesthetized Large-White swine (50.0 ± 4.2 kg) were used in accordance with the European directive 2010/63/EU on the protection of animals used for scientific purposes. All procedures were approved by the French Armed Forces Health Services’ ethics committee.

Swine were about 4-month-old and were housed for a minimum of 5 days in a certified animal facility for acclimation, before being exposed to blast. On experiment days, animals were pre-medicated with an intra-muscular injection of ketamine and xylazine (30 mg/kg, ketamine chlorhydrate, Panpharma, France; 1 mg/kg, Rompun^®^, Elanco France, Ebah, France) and anesthesia was maintained by intravenous perfusion of ketamine (25 mg/kg/h) supplemented with sufentanyl (0.01 μg/kg/h; sufentanyl citrate, Merck, United States) for proper analgesia during surgical procedure. The airway was protected by tracheal intubation, and spontaneous ventilation was preserved. Ventilation efficiency was monitored with EtCO2 (Propaq CS Monitor, Welch Allyn, United States), SpO2, PaO2, and PaCO2 (iSTAT analyzer with CG4+ cartridges, Abbott, United States). Heart rate (HR) and mean arterial pressure (MAP) were monitored using a femoral arterial line. As previously described (Prat et al., 2010), dynamic intra-thoracic pressure impulse was recorded with a Reson TC4013 pressure sensor (Reson A/S, Denmark) positioned into the esophagus. Side-on right costal acceleration was also recorded using a MEMS sensor (PCB, model 3501A12, 60 kG, United States). This uniaxial accelerometer was screwed on the 8th-9th rib (mid-thorax location), counting from the neck to the abdomen. It was fixed so that when the animal is placed in its exposure position, the direction of the shock wave is perpendicular to the sensor. This sensor was used to measure the linear acceleration and the corresponding chest wall velocity by time-integration of the acceleration profile. No correct chest displacement data were obtained due to deviation of the signal from the double time-integration of the acceleration profile. Data were filtered with a 6th order Bessel filter set at 50 kHz.

After blast exposure, animals were kept under ketamine and did not receive any specific care until sacrifice.

#### 2.1.2 Injury Data

Swine were monitored for 60 min after the explosion and then euthanized by exsanguination. Autopsy was performed by a medical examiner. The severity of lung injuries was graded using the pulmonary Axelsson Severity Scale (ASS) (Axelsson and Yelverton, 1996) and the right-to-left lung weight ratio (RL/LL). For ASS, the 0 (negative) to 4 (extensive) scale for lungs was applied.

For RL/LL, after exsanguination, both lungs were weighed and the ratio between the exposed lung weight (RL) on the non-exposed lung weight (LL) was calculated. As both lungs should contain the same proportion of remaining intravascular blood, and because the oedema around the lung injuries was not set yet due to the 1-h observation time, the change (rise) in this ratio is then considered caused by the exposed lung extravascular blood content only.

### 2.2 Blast OverPressure MANnequin: BOPMAN

**FIGURE 1:**
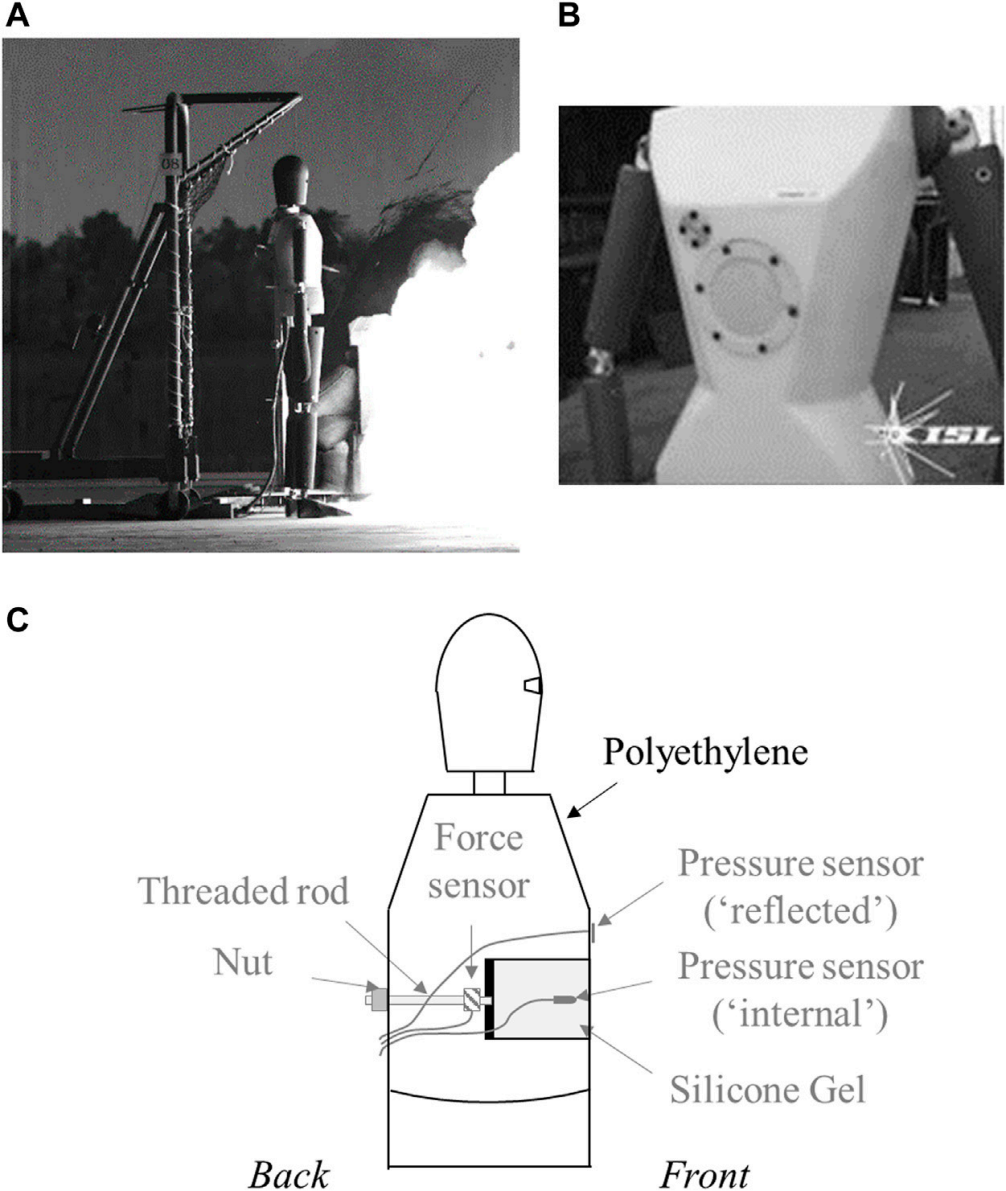
**(A)** Illustration of BOPMAN exposed to a shock wave in standing position; **(B)** Zoom view on the thorax; **(C)** Schematic view of the thorax (side view) with details on the instrumentation.

Figure 1 illustrates the anthropomorphic mannequin BOPMAN measuring 1.86 m for 78 kg. It is mostly made of solid polyethylene, with a specific instrumentation on the thoracic part, as shown in Figure 1C. The center of the thorax is not made of polyethylene but with a kind of drawer filled with silicone gel to represent the soft materials within the thorax. The thoracic part is equipped with:

1) A pressure sensor (Kulite XT190M, 35 bar, United States) allowing the measurement of the reflected pressure on the thorax;
2) A hydrophone (RESON TC4013, Denmark) placed in the silicone gel for the measurement of the internal pressure. It was located at the center of the gel block thanks to a thin plastic support, with the sensor tip located 1 cm behind the front wall of the thoracic part of the mannequin;
3) A force sensor (B&K 8230, 22 kN in compression, and 2.2 kN in traction, United Kingdom) at the rear part of the silicone gel block.

The response of BOPMAN (unprotected) exposed in standing position and in free-field to ideal blast waves of different overpressures and short positive phase durations (<3 ms) can be found in Supplementary Material. As an output of each experiment, the overpressure (ΔP) and the maximum of the impulse (ΔI, defined as the time-integration of the pressure time-history) are obtained from both reflected (subscript “R”) and internal pressures (subscript “int”). The maximum of the force time-history and its maximum impulse are also obtained. All data were filtered with a 6th-order Bessel filter set at 80 kHz.

### 2.3 Experimental Setup

**FIGURE 2:**
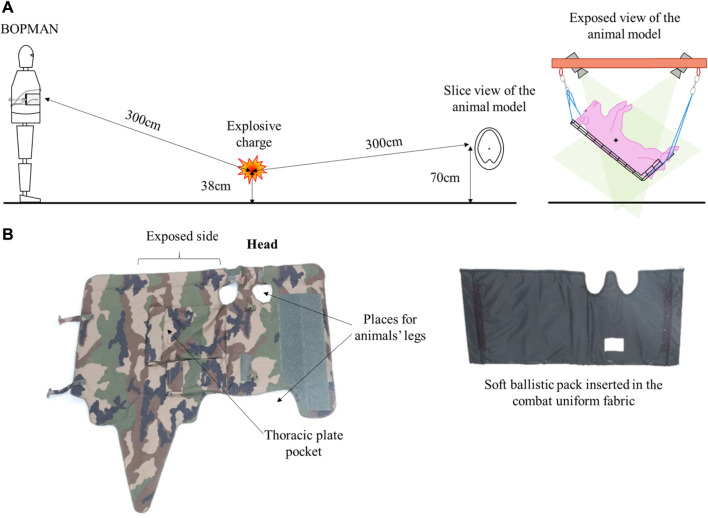
**(A)** Animal and BOPMAN position during a blast exposure (cross on the swine gives the position of the accelerometer); **(B)** Illustration of the animal thoracic protection.

As previously described (Prat et al., 2015), the threat corresponded to a blast overpressure exposure under open-field conditions. Figure 2A illustrates the experimental setup where BOPMAN and the animal were exposed to the chosen blast wave. Only one BOPMAN was used during the experiments since the instrumented part of the mannequin remained undamaged after repeated exposure to blast waves. In order to reproduce an IED scenario, a 4 kg spherical charge was positioned close to the concrete slab (at a height of 38 cm). Animals were placed at 0.65 m from the ground in the Mach stem at a distance of 3 m from the explosive charge, to avoid direct exposure to the fireball. Animals were suspended on a hard net hammock in recline position, their right side facing the explosive charge. As for the mannequin BOPMAN, the instrumented part of the thorax, which is at a height of 133 cm (still in the Mach stem), is at a distance of 3 m from the explosive charge. When equipped with a protection, this is the front face of the armor that is placed at the desire distance relative to the explosive charge. Due to supply issues, the explosive charge used was either a Hexomax formulation with 1.2 TNT equivalence or C4 with 1.37 TNT equivalence in overpressure. The threat was characterized in terms of positive phase duration and peak overpressure level at the target thorax position (and slightly offset to avoid unwanted reflections) by a piezoelectric pencil probe 137A22 (PCB Piezotronics, United States). Physical signals were recorded at a 1 MHz-sampling rate.

Experiments with and without thoracic protective equipment were performed. Animals wearing different types and levels of thoracic ballistic protections sustained the same right-sided open-field blast overpressure. Animals were divided into four groups:

1) A control group (N = 6): not subjected to blast overpressure;
2) P0: no thoracic protection (N = 8);
3) P2: thoracic soft body armor (N = 12);
4) P3: thoracic soft body armor and ceramic hard plate (N = 15).

The vest and soft ballistic pack were specifically designed to tailor the morphology of swine, from the base of the neck to the thighs (see Figure 2B). The density of the P2 protections (soft pack) was 5 kg/m^2^. When combined with the hard ceramic plate for the thoracic P3 protection, the total density attained 43 kg/m^2^. This hard plate was not designed to fit the shape of the swine exposed side and has a curvature. It was positioned in the vest pocket located on the exposed side of swine. The plate could not move in the pocket, therefore, its position relative to the soft pack remained similar for each test. Up to now, there is no standard to confirm the fit of the armor. However, a constant fit of the protection was sought between the different experiments. When the vest was positioned, there was (almost) no gap between the vest and the torso of swine.

When equipped with a TPE, BOPMAN wore the French Army combat suit in addition to either standard P2 (N = 5) or P3 (N = 7) which covers its whole instrumentation. For P0 N = 7. The density of the P2 and P3 protections were similar to those used on swine. Similarly to swine, (almost) no gap existed between the TPE and the thorax of BOPMAN.

### 2.4 Statistical Analysis

Results are given as median [interquartile range]. Statistical analysis was done using Origin Pro software (OriginLab, United States). To compare groups, the Mann-Whitney test was used. A *p* < 0.05 was considered significant.

Data correlations were performed using linear and exponential regressions. R^2^ value was calculated and used to determine the quality of the fit, with a *p* < 0.05 indicating its validity.

## 3 Results

### 3.1 Shock Wave Characteristics

**FIGURE 3:**
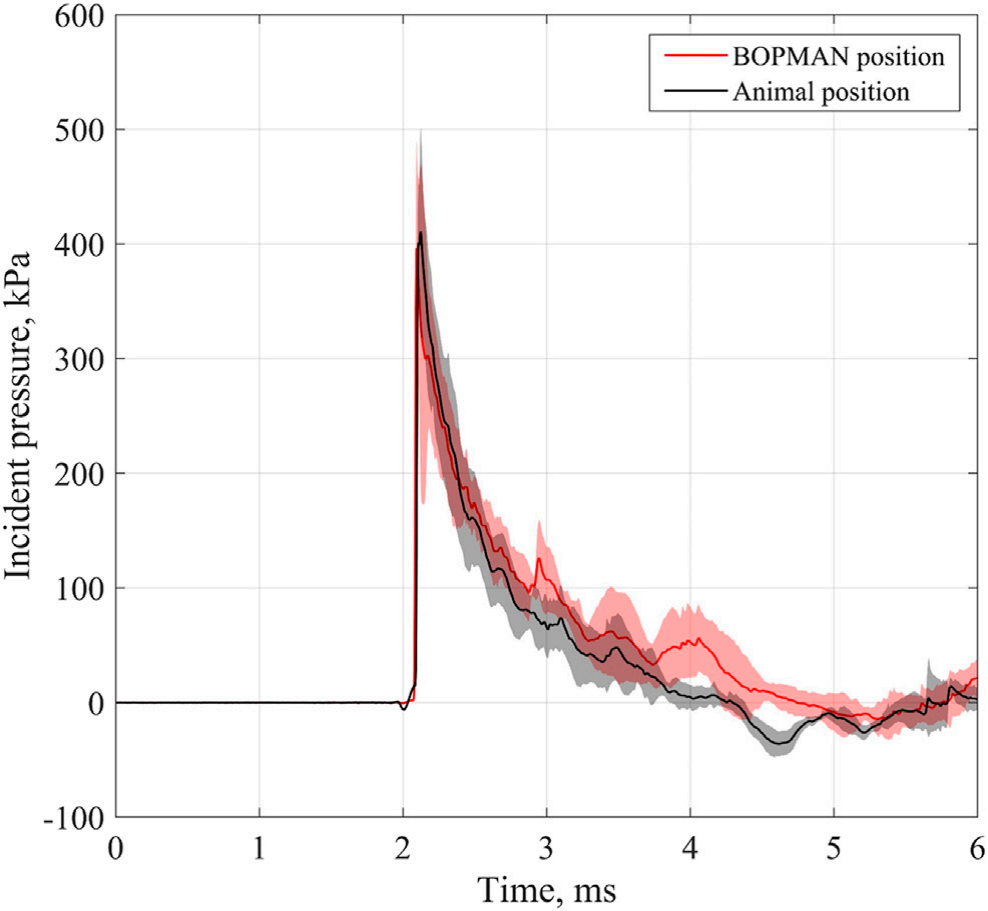
Incident pressure measured near BOPMAN (red line) and near the animal (black line). Solid line represents the mean time history while the shaded area corresponds to the standard deviation.

The characteristics of the pressure-time histories recorded near both thorax locations (the swine and BOPMAN) were close to an ideal Friedlander wave, corresponding to a sudden shock front followed by a near-exponential decay. At the swine level, the median blast wave maximal overpressure ΔP_I_ was 434.5 kPa [417.0–459.5]; its median duration was 2.1 ms [1.9–2.5]; and its median impulse ΔI_I_ was 233.2 kPa ms [220.4–250.0]. At the BOPMAN level, the median blast wave maximal overpressure was 420.3 kPa [403.1–464.8]; its median duration was 2.4 ms [2.3–3.0]; and its median impulse was 223.8 kPa ms [216.7–237.8]. There was no statistical difference between parameters recorded at each location (*p* = 0.72, 0.15, and 0.48 for ΔP_I_, T+, and ΔI_I_, respectively), suggesting that both targets were exposed to similar threats. In both cases, high standard deviations were observed on the positive phase duration (around 20%), which can be explained by the proximity of the targets with the fireball that can lead to disturbances of the shock wave. This similarity in the threat is illustrated in Figure 3, which presents the average (and standard deviation) pressure-time histories measured on both locations.

### 3.2 Animals’ Thoracic Response to Blast

#### 3.2.1 Lung Injuries

**FIGURE 4:**
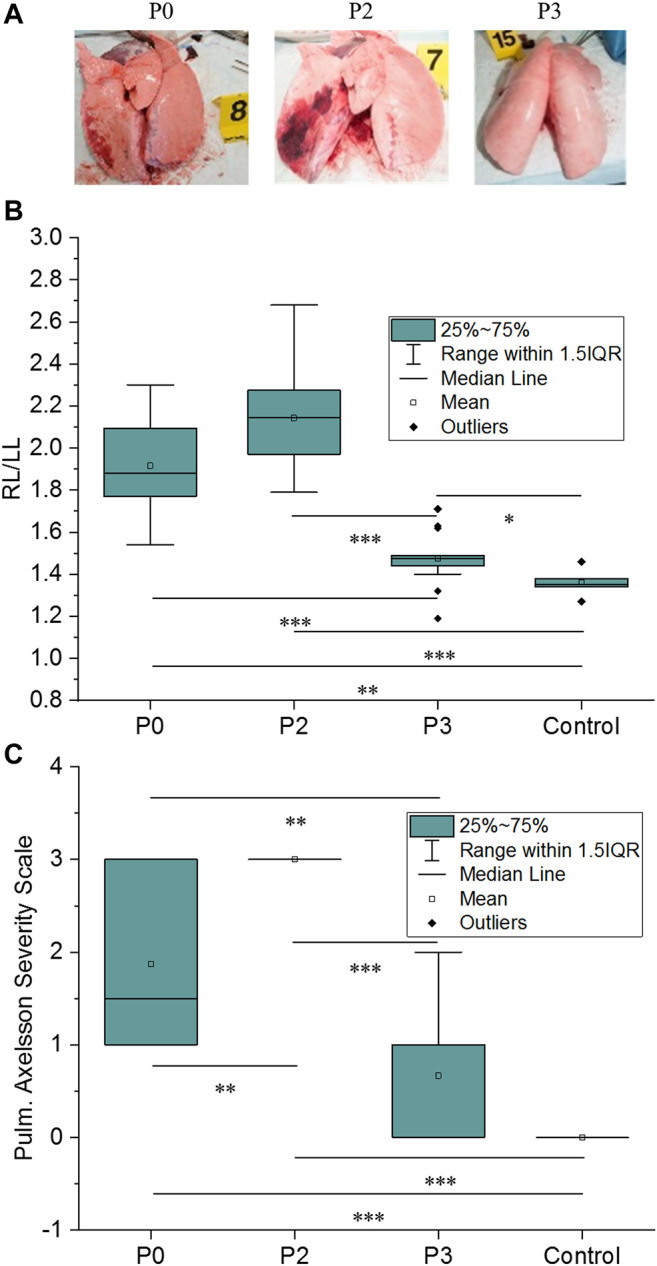
**(A)** Example of lung injuries observed on animals for P0, P2, and P3 groups; **(B)** Right-to-Left lung weight ratio (RL/LL) and **(C)** Pulmonary Axelsson Severity Scale as a function of the protection level. 25∼75% represents the first and the third quartile. IQR is the interquartile range. Median and mean values of the dataset are indicated. *: *p* < 0.05, **: *p* < 0.01, and ***: *p* < 0.001 (Mann-Whitney test).

In our experimental conditions, only the right lung, facing the blast threat, was injured as shown in Figure 4A. For the control group, median RL/LL was 1.35 [1.34–1.38] and was significantly different from P0 [1.88 (1.77–2.10), *p* = 0.002], P2 [2.15 (1.97–2.28), *p* < 0.001], and from P3 [1.49 (1.48–1.71), *p* = 0.032] (Figures 4A,B). The same trend was observed for the pulmonary ASS, which is illustrated in Figure 4C [1.5 (1.0–3.0), *p* < 0.001 for P0, 3.0 (3.0–3.0), *p* < 0.001 for P2, while 1.0 (0.0–2.0), *p* = 0.06 for P3]. The severity of blast-induced lung injuries (Pulmonary ASS and RL/LL) was statistically decreased in animals protected with thoracic ceramic hard plates (P3) as compared to those unprotected (P0) (*p* < 0.001 for RL/LL and *p* = 0.006 for ASS). In addition, there was a trend towards greater lung injury in (P2) animals equipped with soft body armor compared to (P0) unprotected animals (statistically significant for ASS, *p* = 0.007; while *p* = 0.011 for RL/LL).

#### 3.2.2 Thoracic Wall Motion Measurements

**FIGURE 5:**
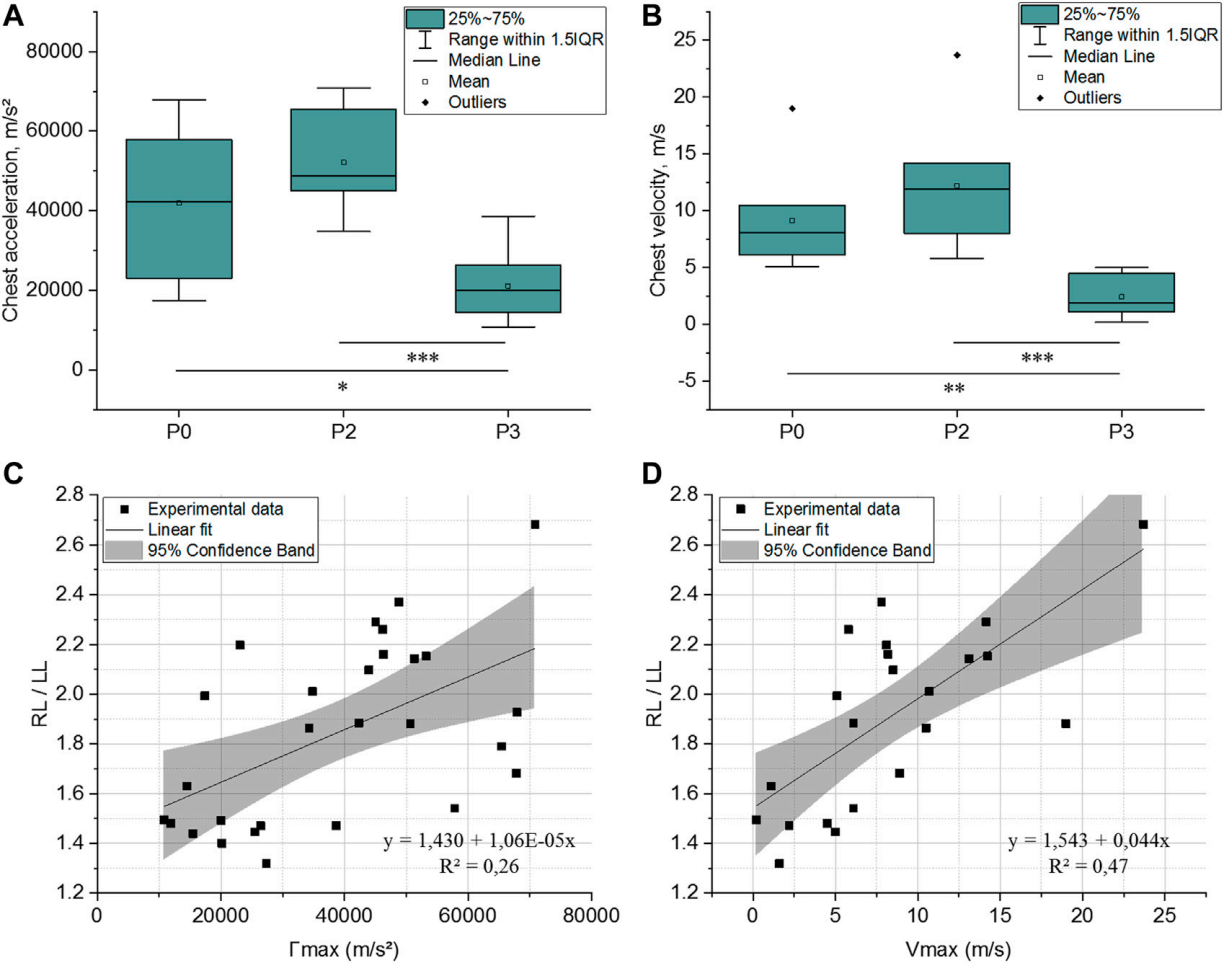
Maximal right thoracic wall costal acceleration **(A)** and velocity **(B)** for the three tested levels of thoracic protection: P0, P2, and P3. *: *p* < 0.05, **: *p* < 0.01, and ***: *p* < 0.001 (Mann-Whitney test); **(C)** and **(D)**: relationship between swine thoracic wall motion level (chest acceleration and velocity, respectively) and lung injury outcomes. Linear fit equations are given on the graphs.

Figures 5A,B display results obtained for maximal right thoracic wall costal acceleration (Γmax) and velocity (Vmax). Both were statistically lower in animals wearing the hard protection (P3), as compared to those unprotected (P0) [20,106 m/s^2^ (14,500–26,414 m/s^2^) *vs.* 42,349 m/s^2^ (23,074–57,827 m/s^2^), *p* = 0.0019 for Γmax and 1.9 m/s (1.1–4.5 m/s) *vs.* 8.1 m/s (6.1–10.5 m/s), *p* = 0.001 for Vmax, respectively]. Despite no statistically significant differences, Γmax and Vmax seem slightly higher in animals equipped with soft body armor compared to unprotected animals [48,793 m/s^2^ (45,035–65,390 m/s^2^) *vs.* 42,349 m/s^2^ (23,074–57,827 m/s^2^), *p* = 0.21 for Γmax and 11.9 m/s (8.0–14.2 m/s) *vs.* 8.1 m/s (6.1–10.5 m/s), *p* = 0.22 for Vmax, respectively].

Figures 5C,D illustrate the relationship between the severity of lung injury (RL/LL) and thoracic wall motion (Γmax and Vmax). Linear fits were applied to both datasets. These fits, given in Eqs. 1, 2, showed a better correlation factor between RL/LL and thoracic wall velocity (Vmax; R^2^ = 0.47, *p* < 0.001) than when RL/LL was correlated to thoracic wall acceleration (Γmax; R^2^ = 0.26, *p* < 0.05).

$$RL/LL=1.430+1.06*10^{-5}\Gamma_{max}\left(m/s^{2}\right)$$ (1)

$$RL/LL=1.543+0.044{\text{V}}_{max}\left(m/s\right)$$ (2)

### 3.3 Thoracic Response of BOPMAN

**FIGURE 6:**
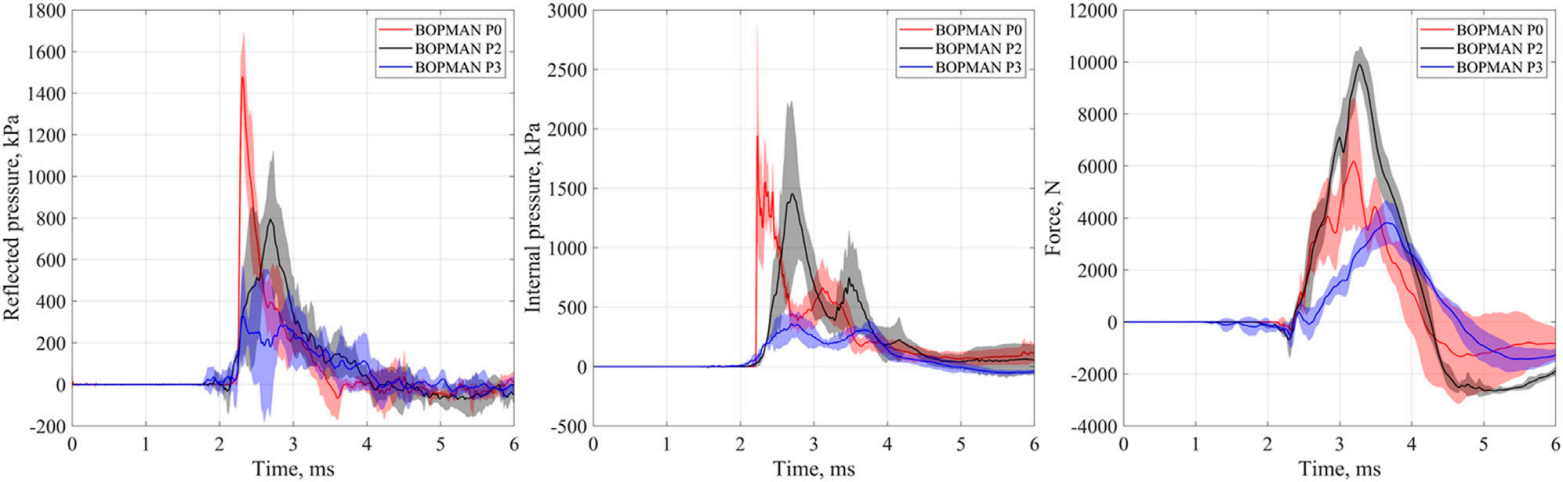
Illustration of BOPMAN measurement for the three levels of protection: unprotected (P0, red line); soft ballistic pack (P2, black line) and hard ballistic pack (P3, blue line). Time histories of the reflected pressure (Left), the internal pressure (middle), and the force (Right). Solid line represents the mean time history, whereas the shaded area corresponds to the standard deviation.

Mean reflected pressure, internal pressure and force-time histories are plotted in Figure 6 for the three protection system levels (P0, P2, and P3). These three parameters do not show the same trend regarding the level of protection system, with either an increase or a reduction compared to the unprotected condition. However, P2 and P3 systematically increased the rise time compared to P0. Two peaks are visible on the internal pressure profile. The first peak corresponds to the arrival of the shock wave on the gelatin block (this is confirmed by the high-speed camera). Looking at the sound wave speed in polyethylene, the second peak could not really be explained by the stress wave traveling from the lower limb to the silicone part through the solid material. Indeed, the shock wave reaches the lower limb at 1.8 ms while it reaches the silicone block at 2.2 ms. Considering a sound speed of 2000–2500 m/s for the polyethylene, the wave would take around 0.6 ms to reach the silicone block. As no contribution are visible on P2 and P3 at 2.4 ms on Figure 6, this hypothesis seems not adequate. Moreover, if it was due to the stress wave propagation in the solid polyethylene, the amplitude would not be influenced by the protection worn. Or, this second peak does not have the same amplitude considering P0, P2, and P3. The second peak could however be due to the roundtrip of the wave in the drawer. The sound wave speed in the silicone part should be measured to validate this hypothesis. But as the time between the two peaks is quite constant for P0, P2, and P3, the hypothesis of the roundtrip of the wave is more plausible. Further, the timing of initial gauge responses is earlier for all of the hard armor conditions. Slight variation around the zero value on BOPMAN measurements when protected can be due to the arrival of the blast wave on the lower part of the protection before it reaches the silicone block.

**FIGURE 7:**
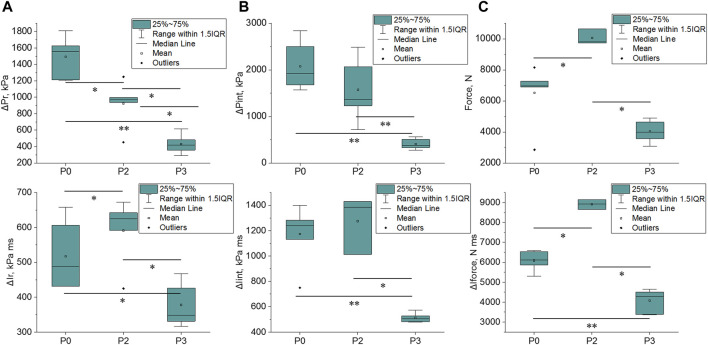
Evolution of parameters measured on BOPMAN regarding wore protection (P0, P2 or P3). **(A)** Reflected overpressure (ΔP_R_) and impulse (ΔI_R_) data; **(B)** Internal overpressure (ΔP_INT_) and impulse (ΔI_INT_) data and **(C)** the force and corresponding impulse (ΔI_FORCE_) data. 25∼75% represents the first and the third quartile. IQR is the interquartile range. Median and mean values of the dataset are indicated. *: *p* < 0.05, **: *p* < 0.01, and ***: *p* < 0.001 (Mann-Whitney test).

Figure 7 illustrates the parameters measured on BOPMAN with any given protection (P0, P2, and P3). The most relevant parameters are those which are influenced by the type of TPE similarly to the level of lung injury measured in anesthetized swine: P3 < P0 ≤ P2. The reflected impulse ΔI_R_, the force and the force impulse ΔI_FORCE_ show this trend. Values from the P3 (hard plate) protected group are lower than those measured from the P0 unprotected group, [348 kPa ms (331–426 kPa ms) *vs.* 489 kPa ms (431–607 kPa ms), *p* = 0.017 for ΔI_R_, 3999 N (3,570–4635 N) *vs.* 6957 N (6,902–7277 N), *p* = 0.065 for Force and 4284 N ms (3,388–4513 N ms) *vs.* 6123 N ms (5,861–6539 N ms), *p* = 0.002 for ΔI_FORCE_, respectively].

Only Force and ΔI_FORCE_ are statistically different between P0 and P2 [6957 N (6,902–7277 N) *vs.* 9794 N (9,727–10,637 N), *p* = 0.024 for Force and 6123 N ms (5,861–6539 N ms) *vs.* 8930 N ms (8,657–9150 N ms), *p* = 0.024 for ΔI_FORCE_, respectively and 489 kPa ms (431–607 kPa ms) *vs.* 625 kPa ms (592–642 kPa ms), *p* = 0.43 for ΔI_R_].

Results from other parameters did not show the same tendencies as the injury severity in living animals (Figure 7).

### 3.4 Relation Between Lung Injury Risk and Measured Parameters on BOPMAN

Only data collected when BOPMAN and animals were wearing the same level of protection and exposed to the same blast wave (ΔI_I_≈230 kPa ms) were included in the following analyses. Data from BOPMAN were correlated to the severity of lung injury observed on animals after postmortem examination. Exponential fits were applied on the data, and demonstrated better fits for the Force and ΔI_FORCE_ (R^2^ = 0.56 and 0.59, *p* < 0.05, respectively) and for ΔI_R_, to a lower extent (R^2^ = 0.14, *p* < 0.05), with the level of injury.

**FIGURE 8:**
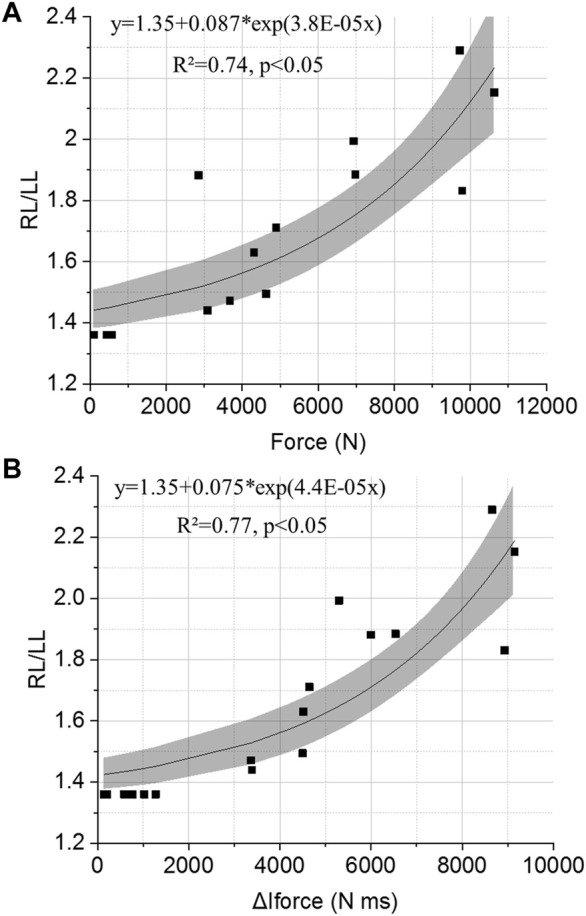
Relationship between the risk of lung injury (right-to-left lung weight ratio, RL/LL) and BOPMAN measured parameters: **(A)** Force and **(B)** ΔI_FORCE_. Exponential fit equations are given on the graphs.

To estimate the risk of injury with BOPMAN for lower blast intensity than the one used during this study, an extrapolation of the exponential fits should be made. Therefore, previously acquired data on unprotected BOPMAN exposed to blast waves which characteristics are known to be non-injurious for the lung according to the Bowen’s curves were used [overpressure (0–100 kPa), duration (0–3 ms), considering the weight adjustment]. As those scenarios are considered not to lead to lung injury, data from BOPMAN were associated with a RL/LL of 1.35, which is the median value of the animal control group. Figure 8 illustrates the relation between the severity of lung injury in blast-exposed animals and the parameters measured on BOPMAN (Force and ΔI_FORCE_). Including these additional non-injury BOPMAN data, exponential fits were applied, and demonstrated good correlations of Force and ΔI_FORCE_ with the level of injury (R^2^ = 0.74 and 0.77, *p* < 0.05, respectively). Fit equations for the Force and the force impulse are given in Eqs. 3, 4, respectively.

$$RL/LL=1.35+0.087e^{3.8*10^{-5}Force\left(N\right)}$$ (3)

$$RL/LL=1.35+0.075e^{4.4*10^{-5}\text{Δ}I_{Force}\left(N\cdot ms\right)}$$ (4)

## 4 Discussion

Contemporary ballistic protections are designed to protect against projectiles. Given the high rate of blast exposure in modern military conflicts, the current study aimed to demonstrate the ability of the anthropomorphic mannequin BOPMAN to evaluate the efficiency of body protections against blast-induced lung injuries.

To achieve our goal, both anesthetized swine and BOPMAN were exposed face-on to a high-intensity Friedlander blast wave (mean characteristics: 434 kPa, 2.3 ms, 236 kPa ms). The present setting was designed to simulate, as close as possible, the primary blast wave generated by an IED exploding at the level of the ground. The recorded peak overpressure and phase duration situated the threat just near the “50% mortality” rate described by Bass et al. (2008) after weight adjustment. Interestingly, this did not result in any death at 1 h post-exposure. This raises the possibility that the assumption made by Bass *et al.* that mortality rates after blast exposure in an open-field and near a wall are similar may be erroneous. By contrast, our scenario leads to 99% survivability on Bowen’s curves, which is much closer to what was observed in our experimental setting. It is somewhat difficult to compare our threat data to Bowen and Bass’s curves, mainly because these curves were based on death rates measured over a 24 h-period, whereas we only measured death rates in our experiments 1 h post-blast. Because blast-induced thoracic alterations may be associated with pulmonary hemoptysis, which can lead to delayed mortality due to progressive asphyxiation, we may have missed few deaths in our animal cohorts. Moreover, bowel injuries can occur and lead rapidly to death such as peritonitis case.

Thoracic injuries measured in our study were consistent with the pattern previously described (Mayorga, 1997), although the right lung only was affected in our experimental conditions. This can be easily explained by the side facing position of the animal in our experimental setting. The lung weight ratio RL/LL was chosen to describe injury severity as it is known to be fairly constant in healthy swine independently of the body weight (e.g., the control group results), and more representative of the extent of lung contusion, given the animals are exsanguinated prior to measurement. Indeed, exsanguination enables to measure intra-alveolar blood accumulation only. Therefore, RL/LL is a much more precise tool for measuring the extent of lung contusion than total lung weight to animal weight ratio. It is noteworthy that blast-induced injuries were restricted to the lung side facing the blast wave in our experimental setting, therefore, abnormal RL/LL data were exclusively due to right lung contusion. We show, here, that NIJ level IV ballistic protection (Ballistic Resistance of Body Armor National Institute of Justice, Standard-0101.06), composed of soft protection and additional ceramic hard plate (P3), provided better lung protection against high-intensity primary blast overpressure than soft protection (NIJ level IIIa, P2). In fact, the level IV protection tested here offered a very good protection against primary blast injuries, as shown by the almost total lack of lung injuries in the P3 group, assessed by ASS and lung weight ratio. By contrast, animals protected by a soft ballistic armor presented lung injuries that were somewhat more severe than those measured in animals with no protection. Previous studies already suggested a possible amplification of the transmitted pressure and an increase in primary blast injuries behind soft body protections (Phillips et al., 1988; Cooper et al., 1991; Gibson, 1994; Cooper, 1996). Indeed, it was demonstrated that multiple plies in ballistic fabric can amplify the peak pressure of the transmitted blast wave, when exposed to short-duration low-intensity blast (Phillips et al., 1988; Cooper et al., 1991; Gibson, 1994) or long-duration high-intensity blast (Cooper et al., 1991). Philips et al. (1988) compared the effects of blast exposure on sheep wearing a soft ballistic vest or no protection and exposed to long-duration low-to high-intensity shock waves. A similar study was conducted by Cooper et al. (1991) on rats and swine which were exposed to short-duration high-intensity blast waves while covered with foam. Overall, these studies demonstrated that lung injury (lung weight/body weight ratio) was increased in animals wearing a low-impedance material, as compared to those that were not wearing any protection. Interestingly, in Cooper and coll. studies, it was demonstrated that, by placing a high-density material between the foam and the incoming blast wave (which is similar to our P3 level protection), a reduction of the level of blast-induced body injury could be observed. It was hypothesized that such an attenuation could result from a “stress-wave decoupling”, which can be achieved by mounting material with a high acoustic impedance (for example the ceramic) onto a material with low acoustic impedance (for example, foam or rubber). It is noteworthy that not all studies to date noticed this amplification phenomenon behind fabric-based armors. Long et al. (2009) exposed anesthetized rats outside a shock tube and concluded that Kevlar vest reduces the mortality rate of the animals. However, rats thorax greatly differs from human’s one, and no indication on the dimensioning of the vest as compared with the size of the animal were given. Wood et al. (2013) also noticed pressure attenuation on a rigid cylinder representing the human thorax when wearing a NIJ II (woven Kelvar vest) and IV protection system (NIJ II + ceramic plate): NIJ IV < NIJ II < unprotected. Reflected pressure from BOPMAN demonstrated a similar evolution (except the NIJ II was here a NIJ IIIa), while swine injury did not follow this trend. This raises the question of the validity of using of the rigid cylinder for evaluation of the efficacy of TPE against blast loading.

**FIGURE 9:**
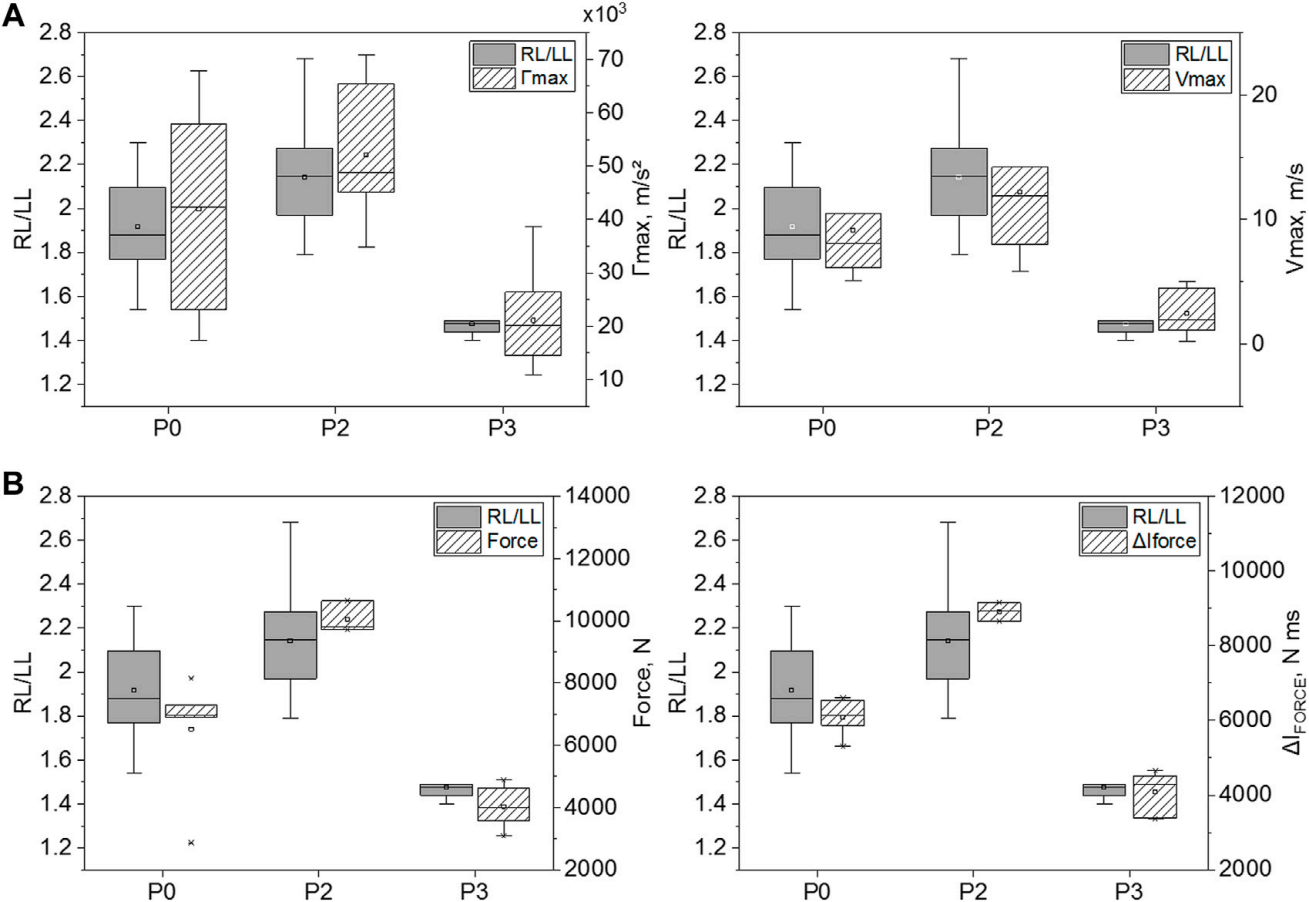
Comparison of the evolution of the lung injury risk for P0, P2, and P3 with **(A)** the swine chest wall motion (chest acceleration and velocity) and **(B)** BOPMAN measured Force and ΔI_FORCE_.

Our study shows that under short-duration high-intensity blast exposure (ΔI_I_≈230 kPa ms), TPE can influence the level of lung injury in swine as follow: P3 < P0 ≤ P2. Chest response to blast was also investigated in swine wearing P0, P2, and P3 level protections. As for lung injury, our results suggest that chest response (costal acceleration and maximum velocity) was distributed as follows: P3<P0≤P2 (Figure 9A). An acceptable correlation fit was found between Vmax and RL/LL (R^2^ = 0.47) in contrast with the Γmax *vs.* RL/LL correlation (R^2^ = 0.26). Chest velocity on swine had previously been correlated with the probability of death in 30 min, but for behind armor blunt trauma (Hinsley et al., 2002). Our result on the chest wall acceleration seems contradictory to Cooper and al. findings (1996) who retained the parameter as injury criterion, with a lung injury threshold defined at 10,000 m/s^2^. Nevertheless, Cooper’s study failed to give details regarding the animal weight, the chest instrumentation, the location of the sensor on the thorax or the sensor fixation procedure, which makes the comparison with our study difficult.

We then investigated whether the mannequin BOPMAN can record the same severity tendencies (P3 < P0 < P2) using its sensors, and evaluate if any parameter measured with BOPMAN is correlated to the severity of lung injury in swine. Data from unprotected (P0) and protected (P2, P3) BOPMAN were analyzed, which led to the conclusion that the force and the force impulse (ΔI_FORCE_) measured at the mannequin thoracic level may be used to discriminate the ability of TPE to reduce or enhance lung injuries (Figure 9B) Indeed, our correlation analyses clearly demonstrated that the force and ΔI_FORCE_ measured on BOPMAN followed the trend P3 < P0 < P2 and that they are highly correlated to the lung injury severity RL/LL in swine (R^2^ = 0.74 and 0.77, *p* < 0.05). Force and force impulse are simply correlated to the observed injury, without any causal relation. This evaluation can only be made for face-on exposure and for a single exposure without any reflection. More data will be needed to validate the use of BOPMAN for multiple exposures or complex blast waves. Existing mannequins have only proven their ability to qualitatively discriminate the level of efficacy of several TPE (better or worse than), but none of them are able to estimate the risk of lung injury, by contrast with BOPMAN. Jetté et al. (2004) studied the response of a torso rig to mild and high-intensity blast without thoracic protection, with low and high impedance materials. Measured maximum thoracic wall acceleration was lower for fragment protective vest and ceramic chest plate (equivalent to our P3 protection) than without protection, while values with the fragment protective vest (equivalent to our P2 protection) were slightly higher than when unprotected. The Hybrid III 50th percentile male mannequin was also used for that purpose (Bass et al., 2005; Carboni et al., 2010). Studies with this mannequin demonstrated that the external thoracic pressure can be reduced by wearing bomb suits, as compared to the unprotected condition (Bass et al., 2005). In addition, these studies found that maximum thoracic wall acceleration was reduced when both thoracic protections (aramid-based fabric with or without hard plate) were used (Carboni et al., 2010). Finally, for short-duration, mild- and high-intensity blast waves, the MABIL mannequin managed to measure higher maximum thoracic wall accelerations with class III protection while lower accelerations were obtained with class IV protection, as compared with data without protection (Ouellet and Williams, 2008). This study showed the evolution of MABIL thoracic wall acceleration as a function of the threat incident overpressure. However, for short-duration waves, risk of lung injury is related to the maximum incident impulse (Boutillier et al., 2019). Therefore, estimating the risk of injury with the MABIL in protected and unprotected settings may not be possible, without further evaluations. Only Cooper et al. (1996b) managed to evaluate the risk for lung injury for high acoustic impedance materials using a rig that simulates the peak of chest acceleration of a swine thorax under blast loading. Nevertheless, no further studies with this rig and no additional data with low impedance materials are available in the literature. Moreover, it used Cooper (1996) chest acceleration criterion with the limitations given in the previous paragraph.

As stated above, animals being hit by the blast wave on their right side displayed ipsilateral, but no contralateral, lung injuries. Bilateral pulmonary contusion was never observed in our experimental conditions. These data, along with those showing that TPE density plays a major role in subsequent injury level, suggest that primary blast injuries to the torso could be regarded as large area direct blunt force traumas, without transmission of a blast wave along tissues. Of course, this hypothesis needs further evaluation.

our study has several limitations. First, the explosive charge used was either a 4 kg spherical charge of Hexomax or C4, which have different TNT equivalence. Nevertheless, change in explosive charge did not impact the overpressure signature curve measured near both models. Then, there are limited data on BOPMAN so far, and it is therefore still uncertain as to whether it could be truthfully used to evaluate the efficacy of TPE for other blast scenarios. Experiments testing different levels of incident impulse should be performed to increase the confidence on BOPMAN’s ability to predict the risk for lung injury in unprotected and protected configurations. Moreover, part of the loading could be missed when equipped with the hard ceramic plate due to the curvature of the plate not perfectly fitting the mannequin shape. If the back face of the protection was in contact with the gelatin part, measurement should include a part due to the wave propagation and a second part due to the impact of the protection on the silicone part. The contribution of each part is unknown and should be studied to evaluate the potential loss of transfer due to the plate curvature. Even if the ceramic hard plate probably does not perfectly fit the swine chest shape (and also the soldier’s), which could lead to different chest motion and lung injury outcomes, the part of the load due to the plate impact will be transferred to the thorax at certain points, contrary to BOPMAN. The fact that a part of the loading could be missed due to the slight spacing between the ceramic plate and BOPMAN silicone block could indeed have an effect on the fitting results (and the relation P3 < P0 < P2) and so the conclusion. If further studies conclude on the importance of the plate impact contribution, the gelatin block of BOPMAN should be modified so that the force recorded at the gelatin block is representative to the mean force applied on the whole plate/thorax area. Furthermore, results obtained on animals may not be readily translated to what is observed in human subjects, because of obvious inter-species morphological differences. A study (Prat et al., 2010) comparing the thoracic wall behavior in large animals and human cadavers submitted to an identical ballistic blunt thoracic trauma showed that the younger pig’s bones were less brittle and more flexible than older PMHS bones, leading to a higher thoracic wall motion under thoracic ballistic impacts. However, comparison to young human subjects is still unknown.

In conclusion, this is the first time, to the authors’ knowledge, that parameters measured on an anthropomorphic mannequin have been correlated to the level of lung injury induced by blast exposure when unprotected or equipped with low and high acoustic impedance body protections. This study provides an initial investigation of using BOPMAN to evaluate the efficacy of TPE against blast loading based on lung injury risk. The results demonstrate that BOPMAN has potential to be used as a test mannequin for evaluating blast lung injury. Additional work to investigate the sensitivity and reproducibility of BOPMAN to other blast exposures (e.g., severity levels, environments) and TPE is necessary to further validate this test device. Validating a test device for evaluating blast lung injury, like BOPMAN, is important to properly evaluate protection systems for soldiers and law enforcement officers.

## Data Availability

The original contributions presented in the study are included in the article/Supplementary Material, further inquiries can be directed to the corresponding author.
