# Supplementary material for: A New Anthropomorphic Mannequin for Efficacy Evaluation of Thoracic Protective Equipment Against Blast Threats
Source: Front Bioeng Biotechnol. 2022 Jan 27;9:786881. doi: 10.3389/fbioe.2021.786881 (PMC8828739; doi:10.3389/fbioe.2021.786881)
Supplement: Supplementary file 1 [file DataSheet1.docx]

Supplementary Material

BOPMAN was exposed in standing position and in free-field to ideal blast waves of different overpressures and short positives phase durations (<3ms). Experiments presented in this supplementary material are from scenarios without thoracic protective equipment (TPE).

As an output of each experiment, the incident blast wave characteristics are obtained with a PCB pencil probe (model 137A22) placed slightly offset from BOPMAN to avoid unwanted reflections and at the thoracic level. From BOPMAN measurements, the overpressure and the maximum of the impulse (defined as the time-integration of the pressure time-history) are obtained from both the reflected and the internal pressure sensors. The maximum of the force time-history and its maximum impulse are also obtained. Figure A1 illustrates BOPMAN measurements regarding the maximum of incident impulse.


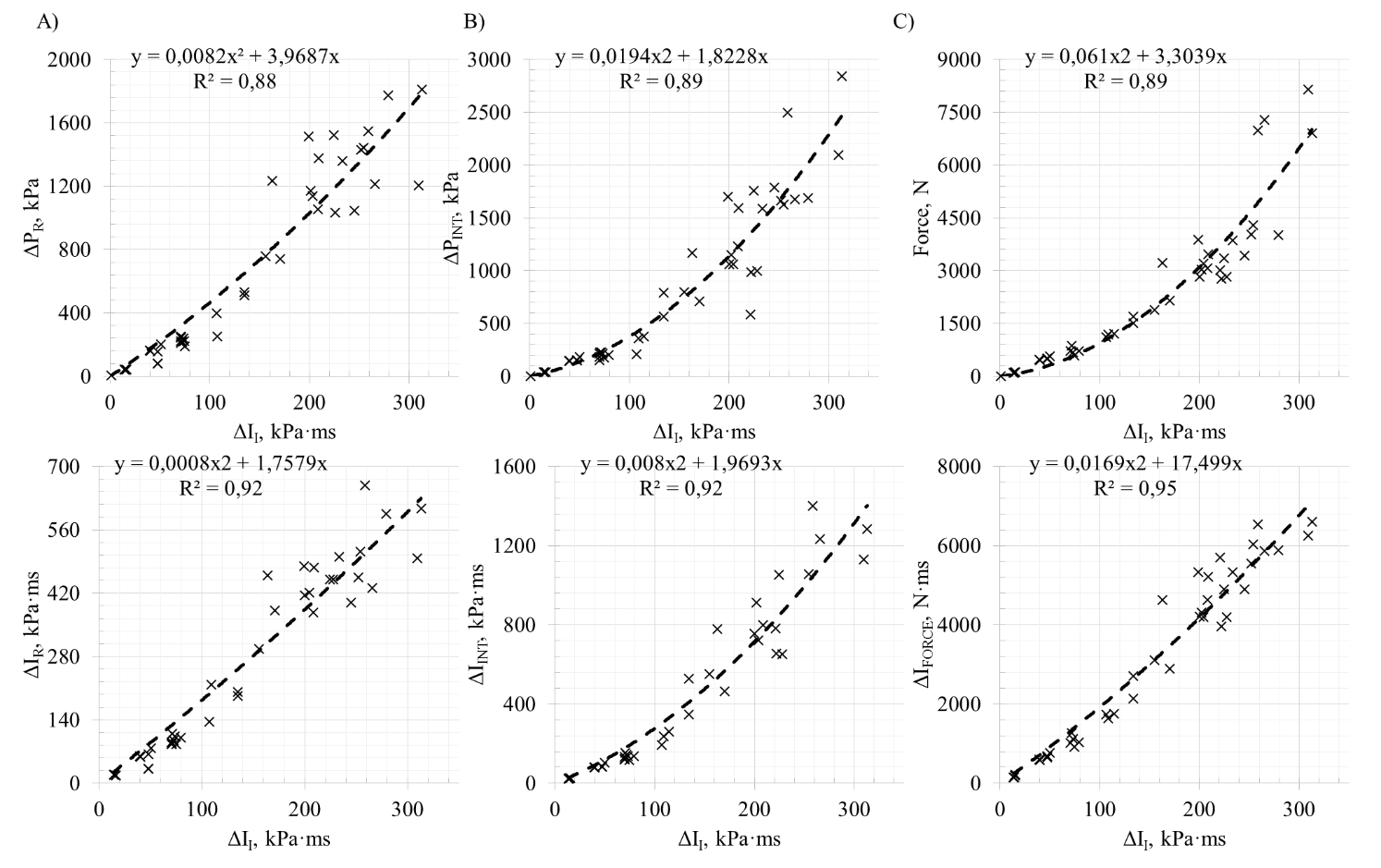


**Supplementary Figure 1.** Evolution of parameters measured on BOPMAN regarding the incident impulse (ΔI_I_). A) The reflected overpressure (ΔP_R_) and impulse (ΔI_R_); B) The internal overpressure (ΔP_INT_) and impulse (ΔI_INT_) and C) the force and corresponding impulse (ΔI_FORCE_).
